# The Association between Baseline, Changes in Uric Acid, and Renal Failure in the Elderly Chinese Individuals: A Prospective Study with a 3-Year Follow-Up

**DOI:** 10.1155/2022/4136373

**Published:** 2022-03-21

**Authors:** Xiuxiu Lai, Bo Gao, Gongmin Zhou, Qingyan Zhu, Yan Zhu, Haijia Lai

**Affiliations:** ^1^Heart Center, Department of Cardiovascular Medicine, Zhejiang Provincial People's Hospital (Affiliated People's Hospital), Hangzhou Medical College, Hangzhou, Zhejiang 310014, China; ^2^Hangzhou Medical College, No. 8 Yikang Road, Hangzhou, Zhengjiang 310059, China; ^3^Department of Prosthodontics, Second Affiliated Hospital of Zhejiang University School of Medicine, Jiefang Road No. 88, Hangzhou, Zhengjiang 310000, China

## Abstract

**Objective:**

The objective is to find whether serum uric acid (SUA) levels are associated with the progression of chronic kidney disease (CKD) remains uncertain, and follow-up data among the elderly population are relatively lacking, especially in China. The aim of the present study was to reveal the association between baseline SUA levels, changes in SUA levels, and renal failure in Chinese elderly adults.

**Methods:**

In this retrospective cohort study, 425 subjects (age range 71–100 years) were analyzed and divided into quartiles based on baseline SUA levels (Q1: <4.8; Q2: <5.7; Q3: <6.5; and Q4: ≥6.5 mg/dl). CKD was defined as estimated glomerular filtration rate (eGFR) < 60 ml/min/1.73 m^2^. We used multiple linear and logistic regressions to compare the risk of renal dysfunction among the different SUA level groups.

**Results:**

The prevalence of hyperuricemia was 24.24% in the elderly subjects. In the multivariable analysis, the odds ratio (OR) for the development of CKD increased with the increase in SUA quartiles at baseline (1.00 *vs.* 1.79 (95% CI, 1.00–3.22), 3.4 (95% CI, 1.79–6.47), and 6.79 (95% CI, 3.45–13.75), respectively; *P* for linear trend <0.001), and a per unit increase in baseline SUA levels gave an OR of 1.76 (95% CI, 1.45–2.14) for renal failure. At the same time, a change in SUA levels had a stronger inverse correlation with a change in eGFR in females (*r* = −0.318, *P* < 0.001) than in males (*r* = −0.187, *P* < 0.01). In a linear regression analysis, a 1 mg/dl increase in SUA levels was associated with an additional 1.25 (95% CI, −1.83 to −0.67) ml/min/1.73 m^2^ decrease in eGFR over a 3-year period.

**Conclusion:**

Elevated baseline SUA levels and changes in SUA levels were associated with a decline in eGFR and an increased risk of CKD in an elderly Chinese population.

## 1. Introduction

Chronic kidney disease (CKD) has become a global public health problem. In China, patients with CKD account for 4.86% of all hospitalized patients, and their median cost and length of hospital stay are higher than those for patients without CKD [1]. Among the end-stage renal disease population, elderly individuals over 65 years constituted the most rapidly growing and the largest age group [2, 3]. The combination of high prevalence and age-related comorbidities might increase the burden on public medical resources. Thus, early detection and intervention for potential risk factors are essential for preventing or delaying the development of CKD and reducing its complications.

Serum uric acid (SUA) is generated from the metabolism of purine nucleotides, and approximately two-thirds of SUA is excreted via the kidneys [4]. Over the last decades, hyperuricemia (HUA) has been considered to be an independent risk factor for the occurrence and progression of CKD [5–8], and uric acid has a “J-shaped” relationship with all-cause mortality in CKD patients [9]. Meanwhile, sufficient evidence has confirmed the clinical benefits of urate-lowering therapy in slowing CKD progression [10]. However, many studies on the association between elevated uric acid levels and CKD have yielded inconsistent results [9, 11]. It is still unclear whether elevated uric acid is simply a marker of renal insufficiency, strongly associated with adverse outcomes in CKD, or both.

Almost all of these studies were focused on adults, and follow-up data among the elderly population are relatively lacking, especially in China. In a cohort study including 1410 elderly residents from Beijing, Zhang reported an adjusted OR of 1.19 in the association between incident CKD and each 1 mg/dl increase in the SUA level [12]. Another study based on a general population in China also showed similar results but with a weak association [13]. In addition, Asians seemed to have a stronger positive association between SUA levels and CKD than non-Asians, despite a lack of statistical significance in the heterogeneity [14]. In short, we aimed to describe the relationship between SUA levels and CKD in an elderly Chinese population-based sample. This study might play an important role in the preservation of renal function in elderly Chinese individuals with hyperuricemia (HUA) and provide clinicians with more knowledge about the risk factors associated with CKD.

## 2. Materials and Methods

### 2.1. Study Design

All participants in this study received two comprehensive health examinations in both 2018 and 2021, which were conducted at the Zhejiang Province People's Hospital. We excluded 38 subjects who had used diuretics or urate-lowering drugs at baseline and 21 subjects for having a past history of the following: a baseline eGFR <15 ml/min/1.73 m^2^; gout; malignancies; acute cardiovascular and cerebrovascular disease; infectious disease; or other life-threatening diseases. Another 29 subjects were excluded due to loss to follow-up. Consequently, a total of 425 subjects (224 males and 201 females, age range 71–100 years) were enrolled in this study. The Zhejiang Provincial People's Hospital Ethics Committee approved the protocol for this study (2020QT179).

### 2.2. Clinical and Laboratory Data

Every subject was required to complete a questionnaire including information on demographic characteristics and medical history. After an overnight fast, they were examined in the morning by trained nurses. The concentrations of SUA, serum albumin, fasting plasma glucose, hemoglobin A1c (HbA1c), hemoglobin, serum creatinine (Scr), blood urea nitrogen (BUN), total cholesterol (TC), triglycerides (TGs), low-density lipoprotein cholesterol (LDL-C), high-density lipoprotein cholesterol (HDL-C), and C-reactive protein (CRP) were measured by using a 717A automatic biochemical analyzer (Hitachi H7600). Urine creatinine (Ucr) and urine albumin (UALB) were measured through an automatic urine analyzer (Sysmex AX4280) and an automatic protein analyzer (Siemens BNII). The urinary albumin-to-creatinine ratio (U-ACR, mg/g) was calculated as UALB/Ucr. Body mass index (BMI) was defined as body weight (kilogram, kg) divided by the square of height (meter, m^2^). Blood pressures were measured by using a standard sphygmomanometer after the subjects rested for 15 minutes.

### 2.3. Diagnostic Criteria

HUA was defined as SUA>7 mg/dl in men and >6 mg/dl in women with a normal purine diet. All subjects were divided into quartiles based on their baseline SUA levels (Q1: <4.8; Q2: <5.7; Q3: <6.5; and Q4: ≥6.5 mg/dl). The estimated glomerular filtration rate (eGFR) (ml/min/1.73 m^2^) was calculated by the CKD-Epidemiology Collaboration (CKD-EPI) equation [15]. CKD was defined as renal dysfunction (eGFR<60 ml/min/1.73 m^2^) [16], and albuminuria was diagnosed if the urinalysis showed a urine dipstick of 1+ or greater, or U-ACR was >30 mg/g [17]. The changes in eGFR and SUA were calculated by the following equations: ∆eGFR = eGFR_2018_-eGFR_2021_ and ∆SUA= SUA_2018_-SUA_2021._ Hypertension was defined as a systolic or diastolic blood pressure ≥140 or ≥90 mmHg, respectively, or the current use of antihypertensive drugs [18]. Diabetes mellitus was defined as a fasting blood glucose level ≥7.0 mmol/l or using antidiabetic medication [19].

### 2.4. Statistical Analysis

All analyses were performed by SPSS v. 22. Continuous variables are reported as the means±standard deviations or interquartile ranges (IQRs), and categorical variables are reported as percentages. Continuous and categorical variables were compared by the *T* test and chi-square test, respectively. Statistical differences among the groups were compared with a one-way analysis of variance (ANOVA) or the Kruskal–Wallis test. In further analyses, multivariate logistic regression analysis was used to calculate odds ratios (ORs) between the baseline SUA level, changes in SUA levels, and CKD, after adjustment for confounding variables. Values with a *P* < 0.05 were used to define statistical significance.

## 3. Results

### 3.1. Baseline Characteristics

The prevalence of HUA was 24.24% in the total population, and the prevalence of HUA was higher in females than in males (27.86% vs. 20.98%, respectively; *P* < 0.001). Among all 425 subjects, the prevalence of diabetes, hypertension, and chronic cardiovascular disease was 36.24%, 80.47%, and 60.24%, respectively.

### 3.2. Levels of Baseline Variables in Each SUA Quartile

Subjects were divided into quartiles (Q1-Q4) by SUA levels at baseline, and the baseline characteristics are presented in Table 1. The mean values of age, BMI, TGs, blood urea nitrogen, serum creatinine, and the prevalence of CKD increased significantly with an increase in SUA levels (*P* < 0.05), while the mean values of baseline eGFR, follow-up eGFR, TC, and HDL-C decreased significantly (*P* < 0.05). Pearson's correlation coefficient showed that the baseline eGFR was inversely correlated with the SUA level at baseline (*r* = −0.371, *P* < 0.001).

### 3.3. Multivariate Regression Analysis of the Association between Baseline SUA, eGFR, and CKD

In Model 1, the OR for the progression of CKD increased as the quartiles for baseline SUA levels increased from the first to fourth quartiles (1.00 vs. 1.72, 3.29, and 5.4, respectively; *P* for linear trend<0.001) (Table 2). In Model 3, this association remained unchanged after adjusting for potential risk factors (age, sex, BMI, TC, TGs, HDL-C, diabetes, hypertension, chronic cardiovascular disease, ACEI/ARB usage). At the same time, the ORs for hyperuricemia and per 1 mg/dl increase in baseline SUA levels for the development of CKD were 3.68 (95% CI: 2.03–6.66) and 1.76 (95% CI: 1.45–2.14), respectively. In the multivariable linear regression analysis adjusted for the above confounders, the baseline SUA levels were inversely correlated with the baseline eGFR (*B* = -3.58, SE 0.43, *P* < 0.001).

### 3.4. Interactive Association of eGFR2018, eGFR2021, ∆eGFR, and ∆SUA

**Figure 1:**
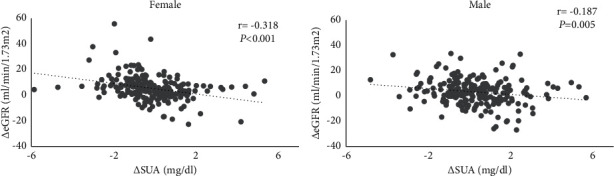
Correlations between changes in SUA levels from 2018 to 2021 and changes in eGFR during the same period in females and males. ∆eGFR = eGFR_2018_-eGFR_2021;_ ∆SUA= SUA_2018_-SUA_2021_.

We performed linear regression analyses to investigate the relationship of changes in SUA levels with declines in eGFR.  Figure 1 shows a significantly negative association between changes in SUA levels and eGFR in both males (*r* = −0.187, *P* < 0.01) and females (*r* = −0.318, *P* < 0.001). After adjusting for confounding factors (sex, age, BMI, TC, TGs, HDL-C, baseline eGFR, diabetes, hypertension, chronic cardiovascular disease, and ACEI/ARB usage), we found that a 1 mg/dl increase in the SUA level over 3 years was connected with an additional 1.25 ml/min/1.73 m^2^ decline in eGFR (Table 3).

## 4. Discussion

We demonstrated that elevated baseline SUA levels and changes in SUA levels were associated with a decline in eGFR and an increased risk of CKD among elderly Chinese individuals after multivariable adjustment, and this association was even observed in the normal range of SUA. In addition, there were sex differences in uric acid levels: women had a higher prevalence of HUA and a stronger inverse correlation in the relationship between the changes in SUA levels and eGFR than men.

In our study, participants in the fourth SUA quartile were 6.79 times more likely to develop CKD than those in the first quartile, and a per unit increase in the baseline SUA level indicated an OR of 1.76 (95% CI, 1.45–2.14) for renal failure. In addition, the OR for CKD significantly increased with baseline SUA levels, even within the normal range (Q2∼Q3, <6.5 mg/dl). This indicated that a mild elevation within the normal range of SUA might be a risk factor for renal dysfunction. Our findings were in accordance with those of previous studies [6,12–14,20]. In a study containing 4,546 volunteers with a 4-year follow-up, Wu N *et al.* determined that the OR for incident kidney disease increased to 2.73 (95% CI, 1.65–4.50) for individuals in the fourth SUA quartile (>5.1 mg/dl) compared to those in the first quartile [14]. Storhaug et al. found that a 1 mg/dl increase in the baseline SUA level was associated with 1.16-fold odds of renal dysfunction after a 13-year follow-up (95% CI, 1.04–1.29) [20]. To our knowledge, the effect value in our study was greater than those in the above experiments [12–14]. The possible reasons for this were age heterogeneity and age-related comorbidities [4], which might interact with uric acid to strengthen the link.

In the analysis of the longitudinal data, we observed that changes in SUA levels were inversely correlated with changes in eGFR, and a 1 mg/dl increase in the SUA level was associated with an additional 1.25 ml/min/1.73 m^2^ decrease in eGFR during the 3-year follow-up after adjusting for possible factors. Consistently, Ye *M* et al. revealed that the time-mean SUA could increase the risk of developing CKD by 6.32-fold [21]. A retrospective study reported a similar result that an increased SUA level indicated a higher risk of deterioration in kidney function, with an adjusted OR of 1.639, while a decreased SUA level helped to slow the deterioration of renal function [22]. In addition to the baseline SUA levels, longitudinal SUA changes were also positively associated with the risk of CKD among middle-aged and elderly Chinese [14]. Additionally, many trials have demonstrated that urate-lowering therapy can delay the progression of renal insufficiency [10]. Thus, when we evaluated the effect of SUA on renal failure at follow-up, we should have taken the longitudinal SUA change into account at the same time, not just the baseline SUA level or a one-time measurement.

Furthermore, we found that sex differences might play an important role in the association. Musacchio *E* et al. revealed that age-dependent UA increases were markedly different in males and females, with the latter showing a steeper trend [23]. In a previous study involving community-dwelling elderly individuals in China, Yang et al. found that women had a higher prevalence of HUA in the age subgroup older than 70 years, which was similar to our results [24]. In our study, women also showed a stronger negative correlation between changes in the uric acid level and eGFR. As we analyzed an elderly population including postmenopausal women, estrogenic hormones that enhance renal urate excretion might be, at least in part, the reason for the discrepancy [25, 26].

However, the link between uric acid and kidney failure remains controversial. In a study by Chang YH et al., uric acid played a pathogenic role only when the value exceeded 6.3 mg/dl, and a lower uric acid level might be beneficial to the improvement of renal function in participants with diabetic nephropathy [27]. Based on a Japanese general population containing 5507 participants, Tada K et al. reported that there was no statistically significant correlation between SUA levels and the progression of CKD [28]. Srivastava A et al. compared the relationship in different CKD stages and found a potentially protective effect between higher UA levels and renal dysfunction in participants with CKD stage 4 or 5 [9]. The conflicting results for the association might be due to differences in study design, including recruited subjects, the calculation of eGFR, the duration of follow-up, and the definitions of outcomes.

A series of experimental studies proposed several possible underlying mechanisms [29–32]: (1) UA crystals may cause direct kidney toxicity by depositing within the kidney; (2) elevated uric acid might induce intrarenal oxidative stress and mitochondrial dysfunction, leading to damage to endothelial cells, smooth muscle cells, kidney tubular cells, and activation of the renin-angiotensin system; and (3) SUA might be a risk factor for metabolic syndrome, diabetes, and hypertension, which could accelerate the development of CKD [32]. Eventually, hyperuricemia might cause glomerular and renal tubulointerstitial injury, leading to a decline in eGFR and albuminuria [33]. However, because of the small sample size and the particularity of the population, we could not clarify the association between uric acid levels and albuminuria.

As a retrospective cohort study, the present study had several limitations. First, the small sample size and the retrospective nature of the study may weaken the potential causality between SUA levels and CKD. Second, we could not eliminate the possible effects of medication that affected uric acid metabolism on the present findings. Third, we had no information about the family history of kidney disease or underlying kidney disease, which should be obtained by renal biopsy and ultrasonography. Finally, the conclusions of this study are not applicable in other ethnic populations because of the sample's demographics.

## 5. Conclusion

This study demonstrated that elevated baseline SUA levels and changes in SUA levels were associated with increased odds of CKD and declining eGFR among elderly Chinese individuals after adjusting for possible confounding factors. Early and appropriate management of SUA levels might slow the development of future declines in eGFR, but we need further large-scale prospective trials to elucidate this issue.

## Figures and Tables

**Table 1:** Baseline characteristics of the participants according to the SUA quartiles at baseline.

Characteristics	SUA quartiles				*P* for trend
	Q1 (*n* = 105)	Q2 (*n* = 110)	Q3 (*n* = 104)	Q4 (*n* = 106)	
Age (years)	85.8 ± 4.1	86.7 ± 3.7	86.6 ± 4.0	87.5 ± 3.7	0.017^*∗*^
Female (n, %)	72 (68.6)	52 (47.3)	45 (43.3)	32 (30.2)	<0.001^*∗*^
SBP (mmHg)	132.3 ± 12.3	130.1 ± 11.9	134.9 ± 13.7	133.6 ± 14.6	0.053
DBP (mmHg)	66.0 ± 8.3	66.3 ± 7.8	68.2 ± 7.9	68.4 ± 8.4	0.061
BMI (kg/m^2^)	23.3 ± 3.3	23.6 ± 3.1	25.0 ± 2.9	24.7 ± 3.6	<0.001^*∗*^
TC (mg/dl)	4.6 ± 1.0	4.4 ± 0.9	4.4 ± 0.8	4.2 ± 1.0	0.018^*∗*^
TGs (mg/dl)	1.1 ± 0.4	1.1 ± 0.5	1.4 ± 0.8	1.3 ± 0.7	<0.001^*∗*^
HDL-C(mmol/l)	1.5 ± 0.4	1.4 ± 0.4	1.3 ± 0.3	1.2 ± 0.3	<0.001^*∗*^
LDL-C (mmol/l)	2.5 ± 0.8	2.3 ± 0.7	2.3 ± 0.7	2.3 ± 0.7	0.349
Fasting plasma glucose (mmol/l)	5.5 ± 1.4	5.5 ± 1.0	5.7 ± 1.5	5.5 ± 1.1	0.578
HbA1c, %	6.2 ± 1	6.1 ± 0.9	6.2 ± 0.8	6.2 ± 0.8	0.843
Hemoglobin (g/l)	127.1 ± 13.9	126.2 ± 14.2	126.9 ± 14.0	126.7 ± 15.9	0.972
Albumin (g/l)	40 ± 4.1	39.4 ± 3.9	40.2 ± 3.8	39.8 ± 4.2	0.546
C-reactive protein (mg/l)	1.4 (0.7,3.1)	1.4 (0.8,3.0)	2.0 (0.9,3.2)	1.8 (1.0,2.9)	0.14
Blood urea nitrogen (mmol/l)	6.3 ± 1.7	6.8 ± 1.7	7.1 ± 1.7	8.3 ± 2.5	<0.001^*∗*^
Serum creatinine (umol/l)	84.2 ± 17.2	93.3 ± 21.2	98.3 ± 19.8	109.5 ± 20.6	<0.001^*∗*^
Albuminuria (n, %)	3 (2.86)	6 (5.45)	9 (8.65)	8 (7.55)	0.312
Baseline SUA (mg/dl)	4.0 ± 0.5	5.2 ± 0.3	6.1 ± 0.3	7.5 ± 0.8	<0.001^*∗*^
Follow-up SUA (mg/dl)	4.6 ± 1.2	5.4 ± 1.0	5.5 ± 1.3	6.4 ± 1.9	<0.001^*∗*^
Baseline eGFR (ml/min/1.73 m^2^)	61.5 ± 11.6	57.9 ± 12.2	54.8 ± 11.2	49.7 ± 11.2	<0.001^*∗*^
Follow-up eGFR (ml/min/1.73 m^2^)	55.7 ± 11.9	53.2 ± 11.9	50.8 ± 12.0	47.2 ± 13.6	<0.001^*∗*^
CKD (n, %)	44 (41.90)	60 (54.55)	71 (68.27)	82 (77.36)	<0.001^*∗*^
Hypertension (n, %)	84 (80)	86 (78.2)	83 (79.8)	89 (84.0)	0.745
Chronic cardiovascular disease (n, %)	63 (60)	66 (60)	62 (59.6)	65 (61.3)	0.995
Diabetes (n, %)	33 (31.4)	43 (39.1)	34 (32.7)	44 (41.5)	0.351
Alcohol intake (n, %)	7 (6.7)	3 (2.7)	5 (4.8)	7 (6.6)	0.515
Current smoke (n, %)	3 (2.86)	0 (0.00)	4 (3.85)	2 (1.89)	0.153
ACEI/ARB usage (n, %)	40 (38.1)	31 (28.2)	43 (41.3)	55 (51.9)	0.005^*∗*^

*Note.* Systolic blood pressure (SBP), diastolic blood pressure (DBP), body mass index (BMI), triglycerides (TGs), total cholesterol (TC), high-density lipoprotein cholesterol (HDL-C), low-density lipoprotein cholesterol (LDL-C), hemoglobin A1c (HbA1c), serum uric acid (SUA), estimated glomerular filtration rate (eGFR), chronic kidney disease (CKD), angiotensin-converting enzyme inhibitors/angiotensin receptor blocker (ACEI/ARB).

**Table 2:** Logistic regression analysis of baseline SUA levels and the development of CKD.

SUA quartiles	Odds ratio (95% confidence interval)			
	Unadjusted	Model 1	Model 2	Model 3
Q1 (<4.8 md/dl)	1.00 (reference)	1.00 (reference)	1.00 (reference)	1.00 (reference)
Q2 (<5.7 mg/d)	1.72 (1.00–2.96)	1.93 (1.09–3.40)	1.82 (1.02–3.25)	1.79 (1.00–3.22)
Q3 (<6.5 mg/dl)	3.29 (1.85–5.84)	3.96 (2.13–7.35)	3.53 (1.87–6.67)	3.40 (1.79–6.47)
Q4 (≥6.5 mg/dl)	5.4 (2.92–9.98)	6.76 (3.44–13.25)	6.43 (3.21–12.87)	6.79 (3.45–13.75)
*P* for linear trend	<0.001^*∗*^	<0.001^*∗*^	<0.001^*∗*^	<0.001^*∗*^
SUA (per 1 mg/dl increase)	1.66 (1.40–1.96)	1.78 (1.47–2.15)	1.74 (1.43–2.11)	1.76 (1.45–2.14)
Normal SUA	1.00 (reference)	1.00 (reference)	1.00 (reference)	1.00 (reference)
Hyperuricemia	3.68 (2.11–6.40)	3.58 (2.03–6.31)	3.41 (1.91–6.09)	3.68 (2.03–6.66)

Model 1 was adjusted for age, sex, and BMI. Model 2 was adjusted for Model 1, TGs, TC, HDL. Model 3 was adjusted for Model 2, diabetes, hypertension, chronic cardiovascular disease, ACEI/ARB usage.

**Table 3:** Linear regression of ∆SUA and ∆eGFR.

	B	SE	SE *β*	95% CI	t	*P*
Model 1	−1.58	0.30	−0.25	−2.17∼ −0.99	−5.26	<0.001
Model 2	−1.25	0.30	−0.20	−1.83∼ −0.67	−4.23	<0.001

*Note.* eGFR: estimated glomerular filtration rate, SE: standard error, CI: confidence interval. Model 1 was adjusted for age, sex, and BMI. Model 2 was adjusted for model 1 plus TGs, TC, HDL, baseline eGFR, diabetes, hypertension, chronic cardiovascular disease, and ACEI/ARB usage.

## Data Availability

The datasets used and/or analyzed in this study are available from the corresponding author on reasonable request.
